# Validation of airway porcine epithelial cells as an alternative to human in vitro preclinical studies

**DOI:** 10.1038/s41598-023-43284-7

**Published:** 2023-09-28

**Authors:** Vincenzo Giuseppe Genna, Davide Adamo, Giulia Galaverni, Fabio Lepore, Federica Boraldi, Daniela Quaglino, Filippo Lococo, Graziella Pellegrini

**Affiliations:** 1 Centre for Regenerative Medicine “Stefano Ferrari”, University of Modena and Reggio Emilia, Modena, Italy; 2 Holostem Terapie Avanzate S.r.l., Modena, Italy; 3 Department of Life Sciences, University of Modena and Reggio Emilia, Modena, Italy; 4 Thoracic Surgery Unit, Fondazione Policlinico Universitario A. Gemelli IRCCS, Rome, Italy; 5 Università Cattolica del Sacro Cuore, Rome, Italy

**Keywords:** Respiratory system models, Cell biology, Adult stem cells, Stem-cell differentiation, Biological models

## Abstract

Animal models are currently used in several fields of biomedical research as useful alternatives to human-based studies. However, the obtained results do not always effectively translate into clinical applications, due to interspecies anatomical and physiological differences. Detailed comparability studies are therefore required to verify whether the selected animal species could be a representative model for the disease or for cellular process under investigation. This has proven to be fundamental to obtaining reliable data from preclinical studies. Among the different species, swine is deemed an excellent animal model in many fields of biological research, and has been largely used in respiratory medicine, considering the high homology between human and swine airways. In the context of in vitro studies, the validation of porcine airway epithelial cells as an alternative to human epithelial cells is crucial. In this paper, porcine and human tracheal and bronchial epithelial cells are compared in terms of in vivo tissue architecture and in vitro cell behaviour under standard and airlifted conditions, analyzing the regenerative, proliferative and differentiative potentials of these cells. We report multiple analogies between the two species, validating the employment of porcine airway epithelial cells for most in vitro preclinical studies, although with some limitations due to species-related divergences.

## Introduction

Animal models have always been used for biomedical research purposes. However, recent evidence highlighted that specific comparability studies could be helpful in understanding when animal models are informative and when they need to be integrated with other in vivo or in vitro assays^1^. Moreover, the adequacy of the animal species chosen as a model should always be carefully evaluated to obtain reliable results from preclinical experiments^2^. Although the employment of animals for research is increasingly controversial^3,4^, their scientific use still remains important, and the optimization of animal experiments is then crucial to obtain reliable results following as much as possible the principle of the three R (replacement, refinement, and reduction). The choice of an animal species as a research model depends on several aspects, including availability, easy handling, size, breeding characteristics and, most importantly, common features in terms of anatomy, physiology and genetics shared with human beings^5^. Small animals such as mice and rats are commonly used to study several human diseases. However, the resulting findings do not always lead to effective clinical applications for human beings, due to differences between the species^6^. Instead, larger animal models, such as swine, show more similarities with the human body structure and better replicate the pathophysiology of certain diseases, resulting in a preferable choice for translational studies, despite being more expensive and complex to manage^2^. Further advantages of using porcine include (i) sharing of several anatomic and physiological aspects with humans and human-size organs^7^; (ii) similar content of genes compared to humans and high protein and gene sequence homologies^8^; (iii) availability of numerous porcine transgenic models to study several human diseases^9^. According to these considerations, swine are increasingly used in biomedical research, for instance in toxicology testing and surgical procedures^10^, infectious disease models^8^, drug discovery research^11^, genetic disease models^12^, xenotransplantation attempts^13^, to end up with bioengineering of transplantable organs^14,15^. Specifically, this animal model has been largely used in respiratory medicine, exploiting the high homology between human and swine airways^16^. For example, many studies on pulmonary surfactants have been performed on porcine lungs^17^, as well as studies focusing on lung development, lung injury induced by hyperoxia or by different toxins, and on several respiratory diseases including asthma, chronic obstructive pulmonary disease, pneumonia, and cystic fibrosis^18^. Moreover, thanks to the longevity of swine, it is possible to evaluate the pathogenesis of respiratory diseases, the long-term efficacy, and the potential adverse events of therapeutic treatments. Like in humans, swine respiratory epithelium changes from proximal to distal airway regions. Indeed, from a histological point of view, porcine trachea and main bronchi are characterized by the presence of a pseudostratified respiratory epithelium, whereas the small bronchi and bronchioles are covered by ciliated columnar epithelium. In addition, unlike smaller animal models as rodents, in humans and porcine numerous submucosal glands are present from trachea to intrapulmonary bronchi^19^. Finally, the heterogeneous composition of the epithelium itself is maintained between human and swine species^20^. The growing evidence of porcine airways’ resemblance to the human counterpart has been followed by an increasing interest in in vitro culture of the respiratory epithelial cells of this animal model. Indeed, in vitro models allow for overcoming some of the main issues related to in vivo animal experiments. For example, they permit to avoid the high costs related to animals’ housing, care, and handling, and to obtain statistically reliable results more easily. Moreover, in vitro experiments ensure a better control of the experimental variables, allow to recapitulate the complexity of the human tissues, and contribute to the reduction of the total number of animals employed during the research, restricting their usage only at the latest stage of preclinical studies. In this view, the great potential of porcine in vitro models has been outlined in several studies including drug screening, infectious disease models, genetic disease studies^21^ and bioengineering-based reconstructive approaches^22^. Furthermore, swine airway samples can be easily obtained as they are usually discarded by-products of slaughtering, avoiding the sacrifice of other animals for experimental purposes only, and the difficulties related to collecting human samples. Importantly, although widely used in biomedical research, few studies compared porcine airway epithelial cells to their human counterpart when subjected to the same in vitro culture stimuli, essential to validate swine epithelial cells as an alternative model to human cells in preclinical studies.

This paper is a comparative study between human and porcine airway epithelial cells from both the trachea and the main bronchi. First, we evaluated the expression and localization of some key molecular markers within the native epithelia. Then we focused on the proliferative, differentiative and regenerative potential of human- and porcine-derived cell cultures under conditions optimized for human cells. The results provided here suggest porcine tracheal and bronchial epithelial cells as suitable alternative cell sources to human cells for in vitro preclinical studies, considering the significant cellular comparability between the two species. However, the observed differences between the two species should be carefully evaluated in future studies, with particular attention to the greater biological variability detected in porcine samples, and the diverse reaction to differentiative stimuli in airlifted conditions. Taking all these aspects into consideration, our findings could not only help strengthen previous studies but also support the selective use of this animal model in the range of preclinical in vitro studies on the respiratory field. Additionally, following the principle of animal use reduction in biomedical research, these studies can be carried out by employing waste materials from the food industry as a source of animal tissues, thus providing an example of a circular economy with a favorable economic impact.

## Results

### In vivo characterization of human and porcine tracheo-bronchial respiratory epithelium

**Figure 1:**
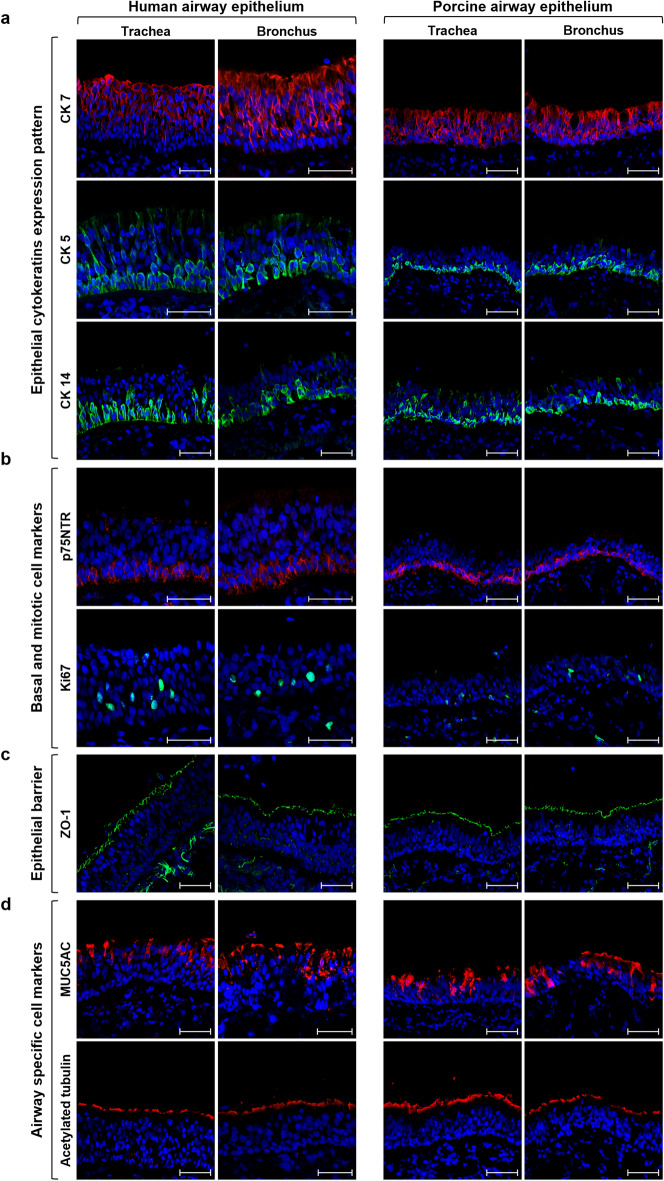
In vivo immunofluorescence analysis of human and porcine airway epithelium. Tracheal and bronchial tissue sections from human and porcine samples were stained for epithelial-specific, proliferation, epithelial barrier and clearance function markers. (**a**) CK7 (red) was mainly expressed in columnar epithelial cells, whereas CK5 (green) and CK14 (green) displayed the expected basal localization in both human and porcine samples. (**b**) Similarly to CK5 and CK14, the neurotrophin receptor p75NTR (red) was mainly detected in both species' basal layer of tracheal and bronchial sections, whereas proliferating Ki67 positive (green) cells, essential for the epithelial renewal, were randomly distributed within the basal and supra-basal layers. (**c**) The respiratory epithelial barrier function was highlighted by the continuous expression of the tight junctions’ protein ZO-1 (green) at the apical level. (**d**) Goblet cells were mainly localized in the sopra-basal layers, producing, storing, and releasing MUC5AC (red), one of the main gel-forming glycoproteins of airway mucus. Moreover, the continuous expression of acetylated tubulin (red) in the luminal side of the tracheobronchial epithelium in both species revealed a high in vivo density of the ciliated cells, essential for a functional mucociliary clearance. Nuclei were counterstained with DAPI, scale bar = 50 µm.

Tissue sections of tracheal and bronchial regions were collected from both species to verify the comparability between human and porcine proximal respiratory epithelium. The expression of structural and functional molecular markers was evaluated through immunofluorescence (IF) assay. The analysis of cytokeratins, cytoskeleton proteins widely used as specific epithelial cell markers, revealed a similar expression pattern in human and pig samples, with cytokeratin 7 (CK7) specifically staining columnar epithelial cells and basal cells identified by cytokeratin 5 (CK5) and cytokeratin 14 (CK14) (Fig. 1a). The similarity between the basal compartment of the two species was also confirmed by the neurotrophin receptor p75 (p75NTR) analysis (Fig. 1b), previously described as a potential lung epithelial basal cell marker^23^. In contrast, the staining for the mitotic cell marker Ki67 highlighted the presence of actively proliferating cells—responsible for airway epithelial renewal—at both basal and supra-basal levels (Fig. 1b). Moreover, the barrier function of the tracheal and bronchial respiratory epithelium was confirmed by the apical expression of the tight junction protein zonula occludens-1 (ZO-1) (Fig. 1c). Finally, both human and porcine samples showed a comparable presence of the two cell populations responsible for the muco-ciliary clearance, namely goblet and ciliated cells, identified by the airway cell markers mucin 5AC (MUC5AC) and acetylated α-tubulin, respectively (Fig. 1d).

### Regenerative and proliferative potential of human and porcine airway epithelial cells

**Figure 2:**
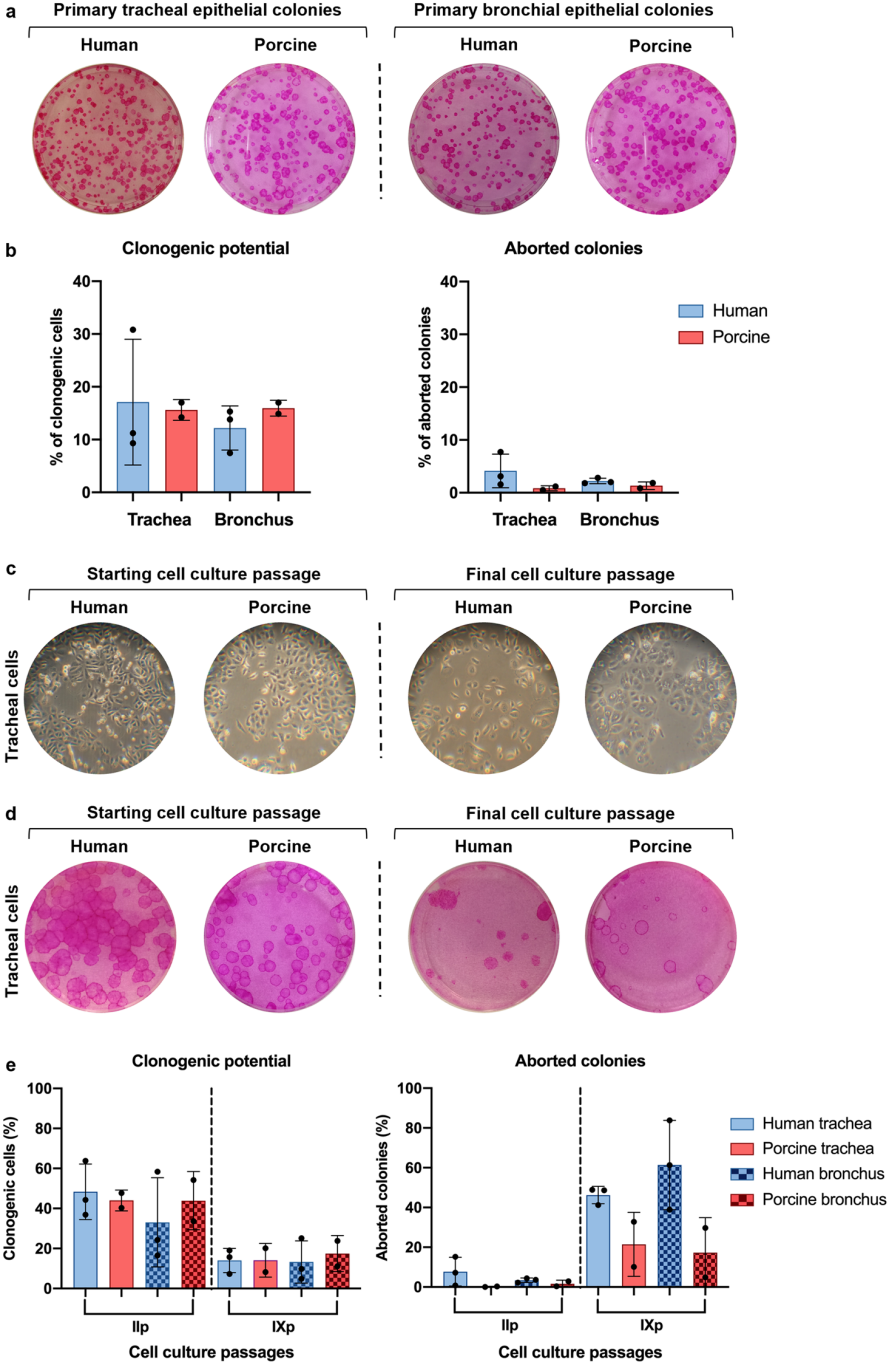
Evaluation of clonogenic potential and cell morphology in prolonged airway epithelial cell cultures. (**a**) Representative images of CFE assay used for the determination of clonogenic and aborted colonies. (**b**) Quantification of the percentage of clonogenic cells (grown colonies, left) and aborted colonies (terminally differentiated colonies, right) respectively out of the total cells seeded and out of the total grown colonies in the indicator dish. No significant differences were observed in clonogenic cells (human *vs* porcine trachea p-value = 0.88; human *vs* porcine bronchus p-value = 0.33) and aborted colonies (human *vs* porcine trachea p-value = 0.27; human *vs* porcine bronchus p-value = 0.20). (**c**) Representative images of human and porcine tracheal epithelial cell cultures at starting (pII) and final (pIX) passages. (**d**) CFE assay evaluates the number of clonogenic and aborted colonies at starting and final sub-cultures. (**e**) Quantification of the grown (left) and aborted (right) colonies in the CFE dishes. No significant differences were observed in the clonogenic potential of human and porcine cells in second (human *vs* porcine trachea p-value = 0.71; human *vs* porcine bronchus p-value = 0.60) and ninth expansion passage (human *vs* porcine trachea p-value = 0.98; human *vs* porcine bronchus p-value = 0.68). Similarly, no significant differences were observed in the aborted colonies percentage in second (human *vs* porcine trachea p-value = 0.25; human *vs* porcine bronchus p-value = 0.25) and ninth expansion passage (human *vs* porcine trachea p-value = 0.07; human *vs* porcine bronchus p-value = 0.10). The results are shown as means ± SD of six human samples (n = 3 trachea and n = 3 bronchi) and four porcine samples (n = 2 trachea and n = 2 bronchi).

The regenerative potential of freshly isolated human and swine primary epithelial cells, namely their clonogenicity, was evaluated by a colony-forming efficiency (CFE) assay^24^ (Fig. 2a) upon processing of tracheal and bronchial biopsies (see Methods). Briefly, this assay evaluates the capability of a single epithelial cell to regenerate a small piece of tissue, namely a colony^25^. Notably, airway epithelial cells directly isolated from the two species displayed a similar clonogenicity in both tracheal and bronchial tissues (human *vs* swine trachea: 17.11% ± 11.92% *vs* 15.61% ± 1.96%; human *vs* swine bronchus: 12.19% ± 4.18% *vs* 15.94% ± 1.50%) (Fig. 2b). Moreover, among the grown colonies, only a tiny percentage (less than 10%) exhausted its proliferative potential (aborted colonies), with no significant differences between human and porcine samples (human *vs* swine trachea: 4.14% ± 3.18% *vs* 0.85% ± 0.48%; human *vs* swine bronchus: 2.23% ± 0.53% *vs* 1.34% ± 0.72%) (Fig. 2b). These findings confirmed the feasibility of efficiently isolating human and porcine airway epithelial cells and pointed out their comparable clonogenic potential.

Human primary epithelial cells progressively lose their proliferative potential upon serial passages in culture until exhaustion, corresponding to replicative senescence. The number of passages in which a decrease in capacity for proliferation is observable depends on several parameters, such as the stimuli of the culture conditions and the intrinsic properties of the primary culture itself^26^. To analyse whether the decrease in proliferative potential displays a similar trend in human and porcine, primary tracheal and bronchial epithelial cells from both species were serially cultivated for seven passages in a commercially available cell culture medium (BEGM™, from Lonza), using the same experimental conditions. Several parameters were assessed, including cell morphology, cell culture yields, population doublings, doubling time, and cultured epithelial cells’ capability to undergo progressive replicative senescence. Curiously, a slight morphological difference was observed between human and porcine cells, with the latter growing in compact clusters rather than independent single cells (Fig. 2c, S1a). Nevertheless, the CFE assay performed to evaluate the clonogenic potential over the passages highlighted a similar trend in both species (Fig. 2d, S1b). In particular, the percentage of clonogenic cells was high in starting passages, with similar values in human and porcine epithelial cultures (human *vs* porcine trachea: 48.33% ± 13.90% *vs* 44.00% ± 5.23%; human *vs* porcine bronchus: 33.07% ± 22.28% *vs* 43.90% ± 14.57%) and progressively decreased in the final passages (human *vs* swine trachea: 14.00% ± 6.03% *vs* 14.15% ± 8.42%; human *vs* swine bronchus: 13.27% ± 10.54% *vs* 17.45% ± 8.98%) (Fig. 2e). Accordingly, the percentage of aborted colonies was low at early passages (human *vs* swine trachea: 7.73% ± 7.21% *vs* 0.15% ± 0.21%; human *vs* swine bronchus 3.40% ± 1.11% *vs* 1.65% ± 1.77%) and increased in the last passages (human *vs* swine trachea: 46.23% ± 4.36% *vs* 21.45% ± 16.05%; human *vs* swine bronchus: 61.33% ± 22.45% *vs* 17.30% ± 17.54%) (Fig. 2e). These results confirmed the capability of both human and swine tracheal and bronchial epithelial cells to undergo senescence over the serial sub-culturing passages, despite the high original proliferation rate.

**Figure 3:**
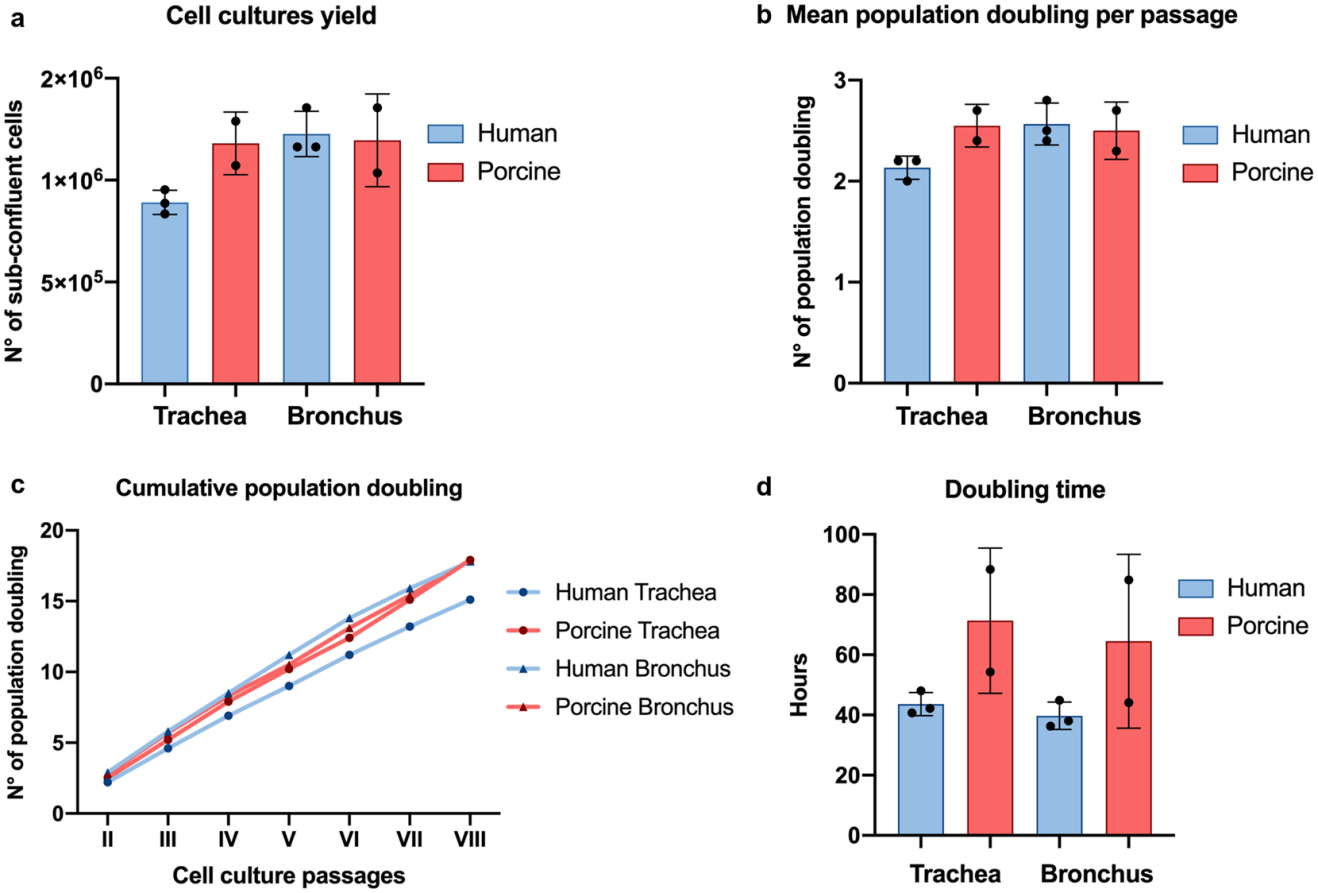
Proliferative potential of human and porcine airway epithelial cells. (**a**) Cell yields average values of human and porcine airway epithelial cells within seven passages of cultures. (**b**) Representation of the mean number of population doublings performed in each passage by human and swine cells. The analysis revealed no significant differences between the two species (human *vs* porcine trachea p-value = 0.06; human *vs* porcine bronchus p-value = 0.78). (**c**) Representation of the cumulative number of population doublings performed by human and swine tracheal and bronchial airway epithelial cells in the seven passages of culture. (**d**) Graphical representation of the average time that the cell cultures take to duplicate. Despite no significative differences were observed in doubling time between the two species (human *vs* porcine trachea p-value = 0.12; human *vs* porcine bronchus p-value = 0.21) porcine cells showed higher biological variability. The data in (**a**), (**b**), and (**d**) are reported as mean ± SD of the investigated parameters for seven passages of six human samples (n = 3 trachea and n = 3 bronchi) and four porcine samples (n = 2 trachea and n = 2 bronchi).

Airway epithelial cells’ growth rate was then analysed by measuring the cell yield at each sub-culture timepoint. In all passages, tracheal and bronchial clonogenic cells from both species generated a similar number of daughter cells, approximately 1 × 10^6^ (Fig. 3a), with also a comparable mean number of population doublings (human *vs* porcine trachea: 2.13 ± 0.12 *vs* 2.55 ± 0.21; human *vs* porcine bronchus: 2.57 ± 0.21 *vs* 2.50 ± 0.28) (Fig. 3b). Indeed, the total number of population doublings measured at the end of the seven passages was analogous in all four respiratory cell cultures (human *vs* porcine tracheal cells: 15.1 *vs* 17.9; human *vs* porcine bronchial cells: 17.8 *vs* 17.9) (Fig. 3c). These data revealed that, in the selected culture conditions, both species’ tracheal and bronchial epithelial cells are endowed with similar clonogenic and proliferative potential. However, although non-significant, a slight discrepancy in doubling time was detected. Indeed, porcine cells showed an higher average doubling time value due to a greater biological variability to reach sub-confluence over consecutive passages than human airway epithelial cells (porcine *vs* human trachea: 71.35 ± 24.11 h *vs* 43.63 ± 3.85 h; porcine *vs* human bronchus: 64.5 ± 28.85 h *vs* 39.73 ± 4.55 h; h = hours) (Fig. 3d).

### In vitro characterization of human and porcine airway epithelial cell cultures

**Figure 4:**
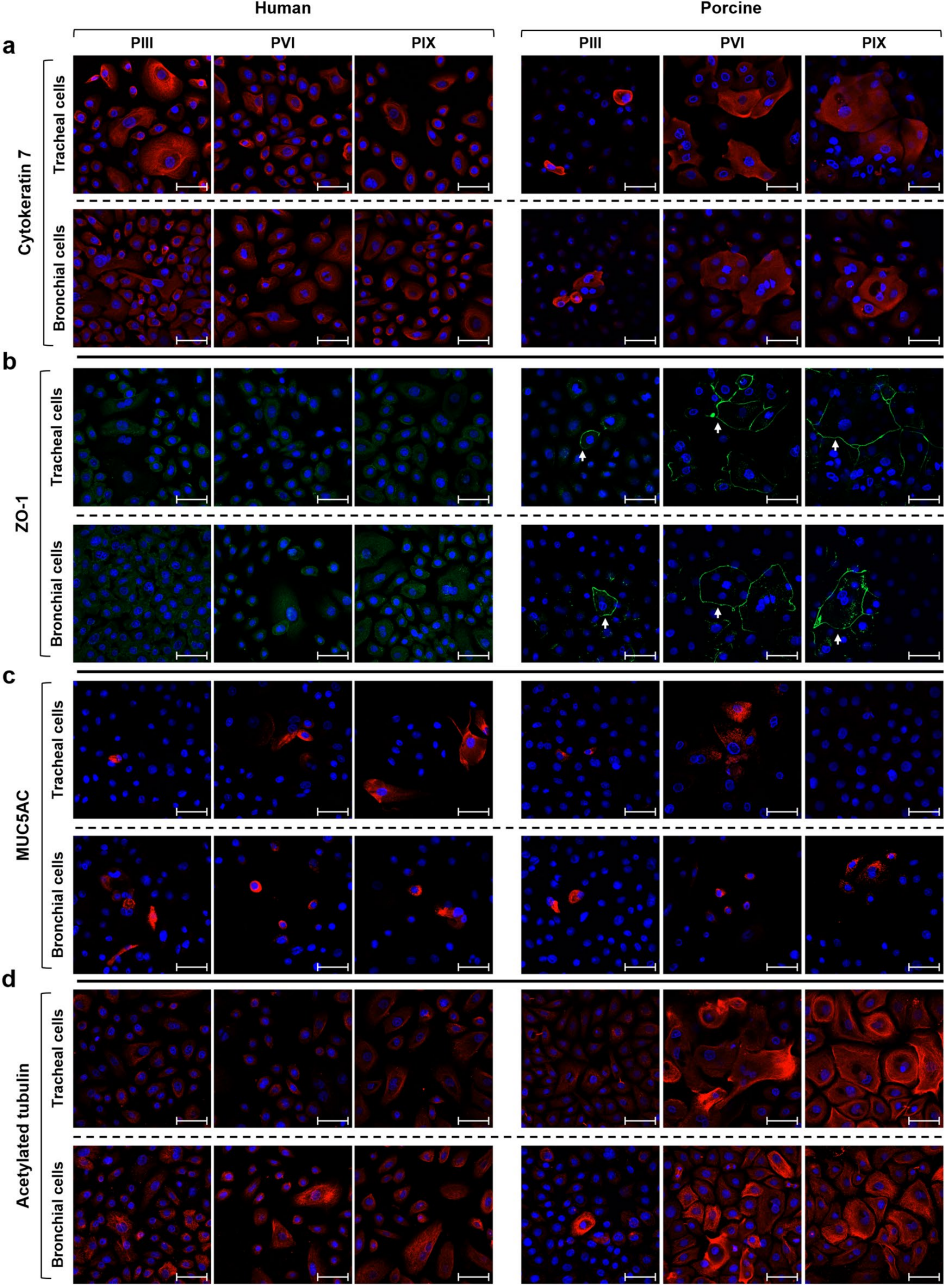
Immunofluorescent analysis of in vitro cultured airway epithelial cells. Human and porcine tracheal and bronchial epithelial cells at the starting (pIII), middle (pVI), and late (pIX) cell culture passages were stained for different markers. (**a**) CK7 (red) was detected in almost all human cultured cells, whereas only some sopra-basal differentiated porcine cells highly expressed this columnar epithelial cell marker. (**b**) ZO-1 protein (green) was barely detectable in human tracheal and bronchial cells, whereas it was expressed by sopra-basal porcine epithelial cells (white arrows). (**c**) Goblet cells identified by MUC5AC glycoprotein (red) were similarly detected in human and porcine airway epithelial cell cultures. (**d**) Within the standard submerged culture conditions, acetylated α-tubulin (red) was expressed at basal levels by almost all cultured human epithelial cells, while it increased especially in middle (pVI) and late (pIX) passages of porcine epithelial cells. Nuclei were counterstained with DAPI, scale bar = 50 µm. p = passage.

The expression of specific identity and function-related cell markers was then analysed at different time points during serial human and porcine cell culture passages, with the aim of evaluating any species-related variations. Airway epithelial cells derived from III, VI, and IX cell culture passages were first stained for epithelial identity markers. In particular, basal cytokeratins, CK5 and CK14, appeared equally expressed throughout the passages, with no differences between human and porcine samples, highlighting the maintenance of basal cells in culture (Fig. S2a). Coherently with previous data on proliferative potential (Fig. 3), the analysis of proliferating cells revealed a similar expression pattern of the mitotic marker Ki67 in human and pig samples (Fig. S2b). In contrast, CK7, expressed mainly by supra-basal cells in vivo (Fig. 1a), was detected in almost all cultured human cells, whereas it was highly expressed only by a few differentiated porcine cells (particularly the large and sopra-basal ones) (Fig. 4a). Moreover, the morphological differences observed between human and swine airway epithelial cell cultures (Fig. 2c) were further reflected by the staining for ZO-1 protein. Indeed, the tendency of porcine cells to grow in more compact clusters resulted in a higher stratification, with an increased expression and an organized localization of ZO-1 tight junction protein (Fig. 4b). Finally, specific airway epithelial cell markers were examined to verify the preservation of the differentiative potential of human and porcine epithelial cultures. In both species, a low number of MUC5AC positive goblet cells was detected throughout the evaluated time points (Fig. 4c). Likewise, acetylated α-tubulin positive cells were observed both in human and porcine cultures, even though these latter displayed a stronger expression in large and stratified cells (Fig. 4d). Generally, the higher ZO-1, CK7 and acetylated α-tubulin staining at late passages of porcine cells could be related to a different colony organization and growth kinetics between the two species when cultured in conditions optimized for human cells (Fig. 4a,b,d).

### The differentiative potential of human and porcine airway epithelial cells

**Figure 5:**
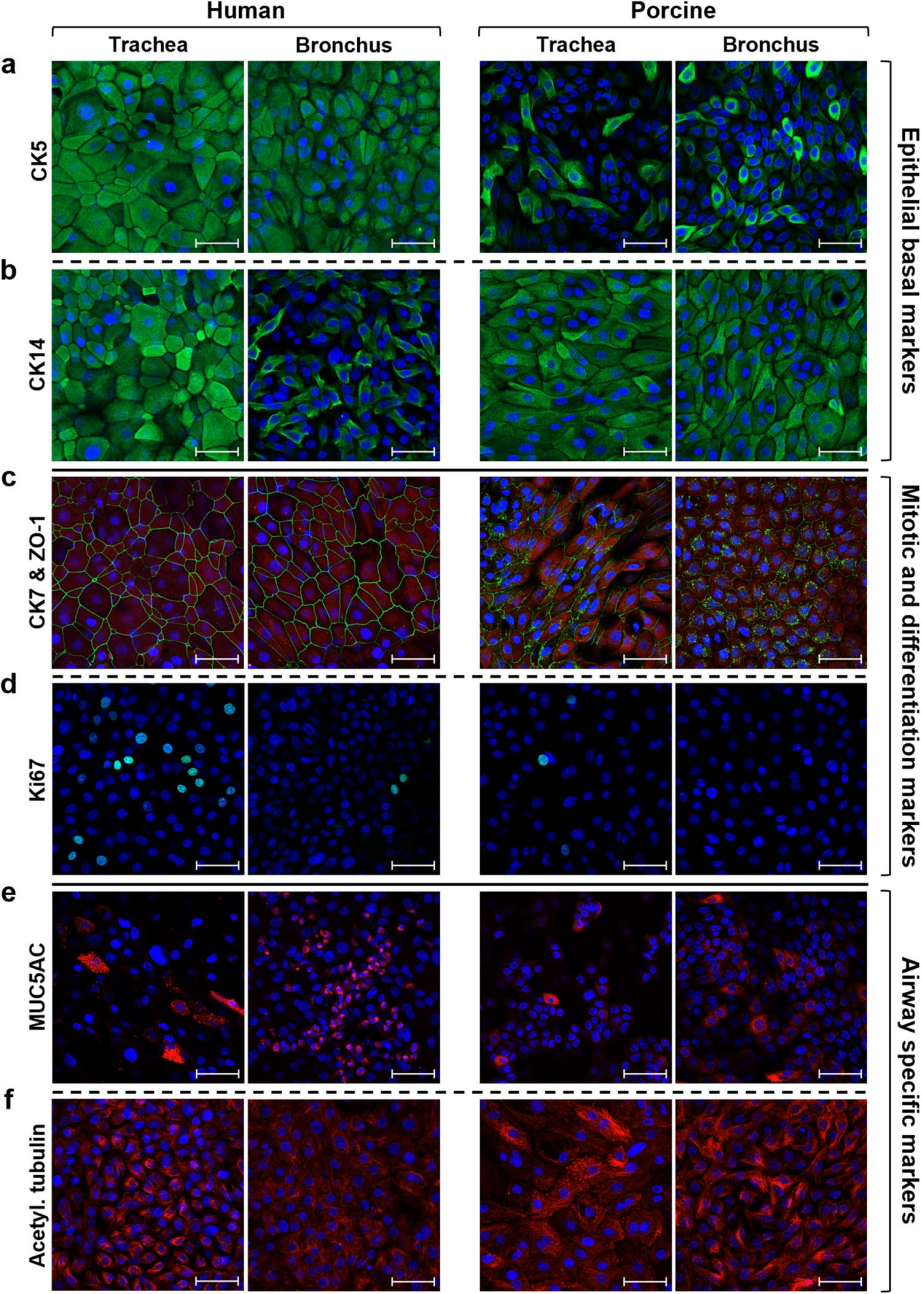
Human and porcine airway epithelial cells in submerged differentiative conditions. IF analysis of human and porcine tracheal and bronchial epithelial cells cultured for 30 days in B-ALI™ differentiation medium. (**a**,**b**) Basal cytokeratin 5 and cytokeratin 14 (green) were expressed at different levels in the submerged stratified cultures. (**c**) All human and porcine cells were CK7 positive (red), while the tightness of the stratified epithelium was highlighted by ZO-1 protein (green). (**d**) Only a few proliferative Ki67 positive cells were detected in all the analysed epithelial cell cultures. (**e**,**f**) The two airway-specific markers MUC5AC and acetylated α-tubulin were expressed at high levels after 30 days in differentiative culture conditions. Nuclei were counterstained with DAPI, scale bar = 50 µm.

Two complementary sets of experiments were conducted to verify whether human and porcine airway epithelial cells could analogously respond to differentiative stimuli. First, tracheal and bronchial epithelial cells were cultured for 30 days in B-ALI™ culture medium under submerged conditions. In contrast, in the second in vitro assay, the cells were exposed to the Air–Liquid Interface (ALI) to better mimic the physiological conditions of the respiratory epithelium. As shown in Fig. 5, several markers were evaluated in submerged cultures to highlight the differentiation stage achievable under this condition. In particular, the basal markers CK5 and CK14 were still detectable after 30 days of culture, although with different expression levels (Fig. 5a,b). Indeed, as previously reported, the expression of these two epithelial cell markers can be differentially regulated during a wound-healing process^27^, which is the condition recapitulated by in vitro epithelial cell cultures. Moreover, during the differentiation process, airway epithelial basal cells change their phenotype toward a stratified tissue architecture, as highlighted by the increased expression of the columnar marker CK7 and the tight junction protein ZO-1 throughout the samples (Fig. 5c), with no differences between human and porcine cells. Furthermore, very few Ki67 positive cells were detected in both tracheal and bronchial differentiated cell cultures at this stage (Fig. 5d), suggesting either a possible exhaustion of proliferative cells or a new homeostatic condition with a balance between proliferation and differentiation. Finally, concerning the airway-specialized epithelial cells, a high number of goblet cells was detected in both species, with the greatest predominance in bronchial cultures (Fig. 5e). Similarly, acetylated α-tubulin appeared highly expressed in 30-days stratified cell cultures (Fig. 5f), although no properly organized cilia were observed. Indeed, as described by Gerovac et al., the submerged culture condition provides a hypoxic environment that prevents a complete differentiation to ciliated cells by inhibiting the gene expression program essential for ciliogenesis^28^.

**Figure 6:**
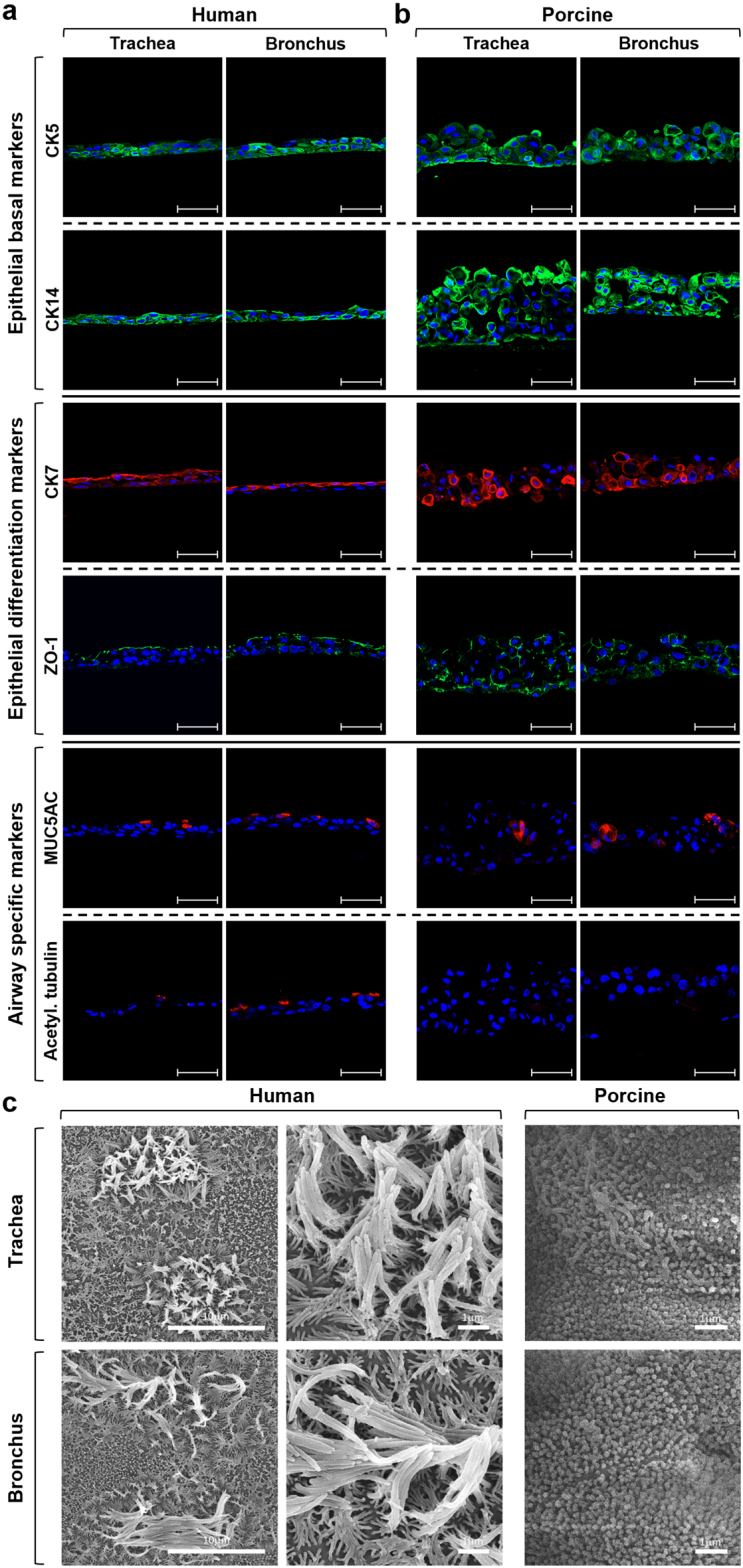
Human and porcine airway epithelial cells in ALI differentiative conditions. IF and SEM analysis of human and porcine airway epithelial cells grown for 30 days in ALI culture. (**a**) Human tracheal and bronchial epithelia appeared well polarized and fully differentiated, as highlighted by the staining for the basal markers CK5 and CK14 (green), by the supra-basal expression of CK7 (red) and by the presence at the apical side of tight-junctions (ZO-1 staining, green), goblet cells (MUC5AC staining, red) and ciliated cells (acetylated tubulin staining, red). (**b**) Conversely, IF analysis of the porcine airway epithelia displayed a less compact structure, as depicted by the staining for CK14 (green) and CK5 (green) and the mislocalization of the epithelial differentiation markers CK7 (red) and ZO-1 (green). Finally, the absence of ciliated cells could be due to the loss of integrity of the swine epithelia in airlifted condition. Nuclei were counterstained with DAPI, scale bar = 50 µm. (**c**) Coherently with IF analysis, SEM images of human tracheal and bronchial ALI cultures showed well-differentiated epithelia with ciliary structures, while porcine tracheal and bronchial epithelia SEM images show the presence of apical microvilli only.

Therefore, to allow a physiological polarization and differentiation, human and porcine airway epithelial cells were cultured in ALI conditions. Human tracheal and bronchial epithelial cells could stratify and regenerate a tightened and fully differentiated airway epithelium, as highlighted by the staining for the basal markers CK5 and CK14, by the supra-basal expression of CK7 and by the apical presence of tight-junctions, goblet and ciliated cells (Fig. 6a). Noteworthy, as observed by the transmitted light microscope, the porcine epithelia revealed the loss of confluence and structural integrity between days 20 and 30 of culture (data not shown). Indeed, at the end of the ALI culture, the porcine airway epithelia displayed a less compact structure, as depicted by the staining for CK14 and CK5, with the mislocalization of the epithelial differentiation markers, CK7 and ZO-1, and the absence of ciliated cells (Fig. 6b).

These findings could be related to a diverse differentiation process and growth kinetics in the two species when cultured under ALI conditions optimized for human cells, affecting the tissue organization.

To further confirm these data, the epithelia of both species were analyzed by scanning electron microscopy (SEM) after 30 days of differentiation. As expected, only human tracheal and bronchial airway epithelia appeared well differentiated and characterized by ciliary structures (Fig. 6c, left). Conversely, the loose cell–cell interaction of porcine cells was also observed during SEM preparation, where porcine specimens appeared non-ciliated and covered by apical microvilli (Fig. 6c, right). This observation confirmed that, after prolonged ALI culture conditions, the porcine epithelium does not show terminal differentiation, likely due to the loss of integrity.

### Trans-epithelial electrical resistance assessment

**Figure 7:**
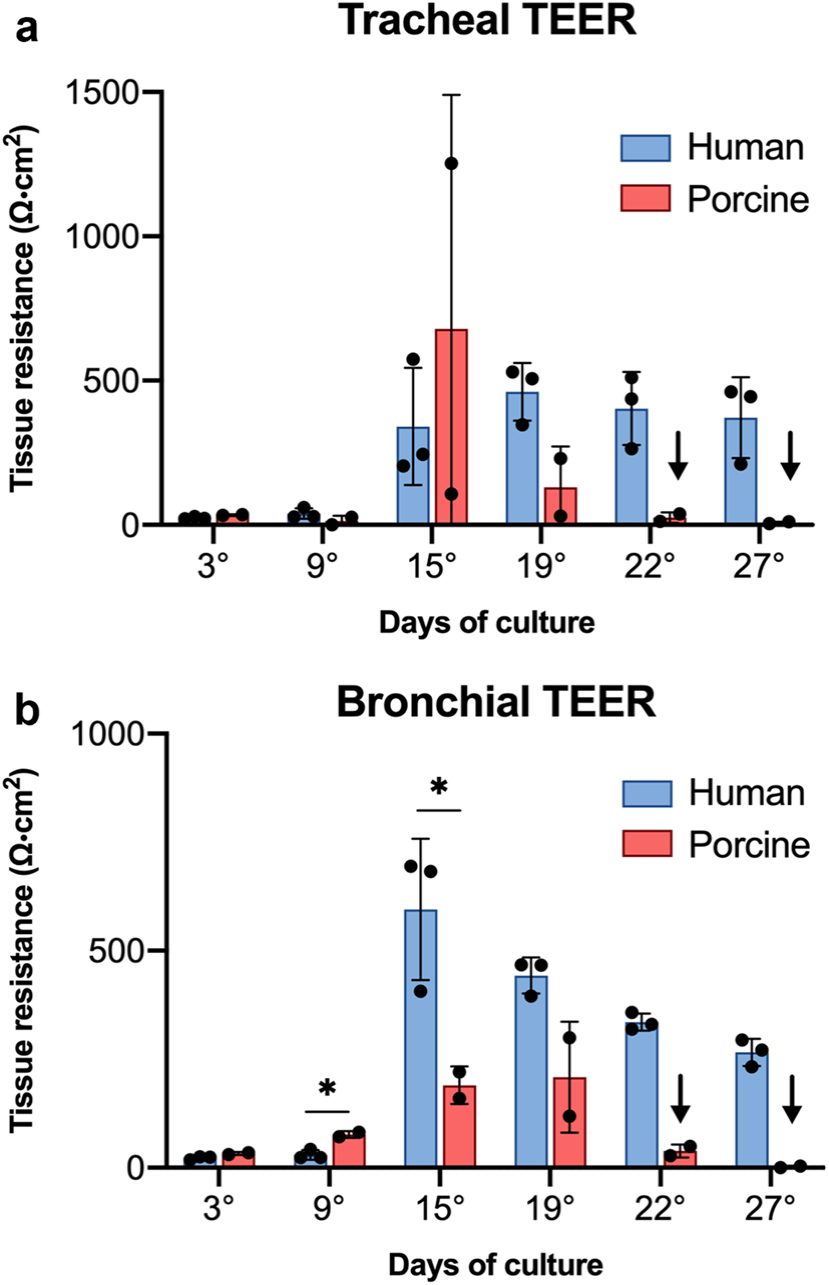
Trans-epithelial electrical resistance measurement. Unit area resistance was measured at six different time points in (**a**) tracheal and (**b**) bronchial human and porcine ALI cultures. Both tracheal and bronchial cultures were characterized by an initial TEER increase in accordance with the differentiation process, although only human cells maintained elevated values. No significative differences were observed in tracheal cultured tissues between the two species (human *vs* porcine p-value = 0.06 at day 3, 0.21 at day 9, 0.51 at day 15, 0.051 at day 19). In bronchial cultured tissues, significative differences were observed only at days 9 and 15 (human *vs* porcine p-value = 0.014 at day 9, 0.047 at day 15), whereas no differences were detected in the other time-points (p-value = 0.06 at day 3 and 0.051 at day 19). Results are expressed as the mean ± SD of the unit area resistance (n = 3 for humans; n = 2 for porcine). Arrows highlight the two time points (days 22 and 27) where porcine epithelia lost structural integrity. Coherently, for these measurements the statistical analysis was not carried out. * = p < 0.05.

To verify if the expression of the ZO-1 protein, previously described in Figs. 5 and 6, was associated with a functional barrier role, Trans-Epithelial Electric Resistance (TEER) measurement was performed at six-time points in human and porcine ALI cultures. Human tracheal epithelium reached a maximum mean value of 460 Ω cm^2^ on day 19 and remained almost stable till the end of the culture. In contrast, porcine tracheal cells reached the highest average value of 680 Ω cm^2^ on day 15 but quickly dropped in the subsequent measurements (Fig. 7a). The human bronchial epithelial cells showed a maximum value of about 600 Ω cm^2^ on day 15 with a moderate decrease in the following days. In reduced to almost zero on the last two time points (Fig. 7b). The increasing values of resistance registered in the first four measurements suggested the capability of both human and porcine airway epithelial cells to properly stratify and differentiate into a mature and tight epithelium, able to perform its function as a barrier. By contrast, the subsequent decrease observed in porcine cultures would suggest the need for ALI culture conditions more specific for porcine cells to obtain a stable TEER on the long term, as with human tissues.

## Discussion

Using animal models in biomedical research is instrumental for understanding the pathophysiology of human disorders and performing preclinical studies, including safety and efficacy tests of new drugs, surgery and therapies. In airway-related research, several animal models have been used to study the most common human respiratory pathologies, including pneumonia, asthma, chronic obstructive pulmonary disease, pulmonary fibrosis, hypertension, and genetic disorders^18^. However, anatomic, physiological and genetic differences between laboratory animals and human beings still represent a critical limit. The most striking example of this phenomenon was observed in cystic fibrosis, a genetic disease where the most common genetic mutation ΔF508 in the Cystic Fibrosis Transmembrane Regulator (CFTR) gene does not cause in mice the respiratory abnormalities observed in humans^29^. Therefore, a comparative analysis between humans and animal models appears critical to understand the species-related limits and to better focus on those similarities allowing the obtainment of meaningful and reliable results. Swine are increasingly used in airway biomedical research for their comparability to humans regarding anatomical, physiological, and genetic features^10,16^. Concurrently, the use of alternative models such as cell cultures, organoids and organ on chip is gaining more and more importance. Indeed, Stennert and colleagues showed that porcine respiratory epithelial cell cultures share with the human counterpart comparable cilia beat frequency and a similar reaction to different drugs, suggesting porcine cell cultures as a useful model for the specific evaluation of pharmacological effects on the respiratory mucosa^30^. Moreover, primary swine tracheal epithelial cells were seen capable of producing inflammatory cytokines (TNFα and IL-1β) when exposed to toll-like receptor agonists, confirming their usefulness also for host–pathogen interaction evaluations^31^. Moreover, several authors took advantage of porcine epithelial cell cultures to investigate airway epithelial cells infection by different pathogen agents such as influenza virus and moulds^31–33^. Here, we focused on the assessment of similarities and differences between humans and swine airway epithelia at cellular level. To identify where the airway porcine epithelium recapitulates the human one, we compared the two species assessing the expression of specific markers in in vivo tissues as well as the proliferative, differentiative and regenerative properties in vitro. Indeed, such analyses are of pivotal importance to validate the swine respiratory epithelium as a comparable in vitro model to the human counterpart.

Coherently with previous publications^20^, the characterization of human and porcine tracheal and bronchial tissue in vivo confirmed a similarity in tissue organization across the two species. Moreover, the evaluation of epithelial airway-specific molecular markers on in vivo samples allowed the identification of culture conditions permissive for the preservation of the cell heterogeneity typical of the respiratory epithelium. Indeed, the analysis of cultured human and porcine cells revealed the maintenance of basal cell markers (CK5 and CK14) expression as well as the capability to properly differentiate toward both tightened sopra-basal cells (CK7 and ZO-1 positive cells) and airway-specialized cells (goblet cells). A slight difference in cell–cell interactions was observed in human and porcine cultures during the early stage of expansion, as swine cells showed the tendency to grow in compact clusters, whereas human cells grew as isolated spindle-shaped cells. Focusing on the growth potential of cultured epithelial cells, a similar clonogenicity of cells isolated from both species suggested a comparable regenerative potential of airway epithelial cells freshly isolated from biopsies. Moreover, through serial in vitro sub-cultures, other significant similarities emerged, including an analogous clonogenic and proliferative potential in terms of cell yield and population doublings between human and porcine tracheal and bronchial epithelial cells. Finally, the analyses conducted under 30 days of extensive differentiation in submerged or ALI conditions highlighted variations in the differentiative process between the two species. The cells from both species could regenerate a fully stratified and polarized tissue characterized by a dense network of tight-junctions (ZO-1 protein) and the airway-specialized goblet cells. These observations are consistent with what was previously described in the literature^30,34^. However, the ciliated cells were adequately detectable only in human ALI cultures, whereas the porcine cells lost the tissue integrity and did not show this terminal differentiation lineage.

In addition, as reported by other research groups with different experimental conditions^30,32,34,35^, the functionality of the described tight junctions both in human and porcine samples was confirmed, yet with some differences, by TEER measurements. Indeed, the barrier role of human and swine regenerated epithelia was demonstrated by the maximum resistance peak reached around the 15-20th day of culture. After this period, the loss of swine epithelial integrity associated with a drastic decrease of the corresponding TEER values opposes to a gradual reduction of human epithelia resistance. This phenomenon highlights the higher capacity of human epithelial cultures to maintain the tissue integrity under extensive differentiation stimuli. Conversely, the degeneration of the porcine epithelia after a prolonged ALI exposure may denote that the culture conditions selected to guarantee the early adhesion, spreading and proliferation of cells from both species were not permissive for the long-term regeneration of a fully differentiated airway porcine epithelium. In this view, the regeneration of a stable fully differentiated porcine epithelium could require the standardization of culture conditions specifically optimized for swine cells, as reflected by the plurality of experimental protocols involving isolation techniques, culture media, and coating matrices^30–34^. Overall, this comparative study pointed out similarities and divergencies between human and swine airway epithelium (Table 1). Specifically, in the selected culture conditions, submerged porcine airway epithelia can recapitulate the human ones in terms of proliferative and differentiative potential, thus representing a suitable alternative to perform specific preclinical studies, including toxicological and pharmacological testing.

**Table 1:** Similarities and divergences between human and swine airway epithelium.

Parameters under comparative analysis	Similarity	Divergency
In vivo tissue (Fig. 1)	✓	
Clonogenicity of biopsy-derived cells (Fig. 2)	✓	
In vitro cell morphology and colony organization (Fig. 2; Fig. S1)		✓
Clonogenic potential in serial passages (Fig. 2; Fig. S1)	✓	
Cell culture yield (Fig. 3)	✓	
Population doubling (Fig. 3)	✓	
Doubling time (Fig. 3)	✓	✓
In vitro cell culture characterization (Fig. 4; Fig. S2)	✓	✓
Differentiative potential: 30 days submerged tissue (Fig. 5)	✓	
Differentiative potential: 30 days airlifted tissue (Fig. 6)	✓	✓
Trans-epithelial electric resistance (Fig. 7)	✓	✓

## Methods

### Human and porcine specimens

Human tracheal and bronchial specimens were obtained in accordance with the tenets of the Declaration of Helsinki; donors (35–70 years-old) provided informed consent for biopsies and ethical committee approval was obtained from the involved centres (“Comitato Etico Area Vasta Emilia Nord”, Protocol N. 2019/0014725 and “Comitato Etico Territoriale Lazio Area 3” N. 0008968/21). Porcine tracheal and bronchial specimens were obtained by 9–10 months-old pigs as by-product from the slaughterhouse, while Normal Human Bronchial Epithelial cells (NHBE) were purchased from Lonza.

### Humans and porcine airway epithelial cell culture

Tracheal and bronchial epithelial cells were isolated from human and porcine biopsies and cultivated on plastic cell culture plates using the commercially available Bronchial Epithelial Cell Growth Medium (BEGM™, Lonza), specifically defined for human primary bronchial epithelial cells’ growth. Briefly, tracheal and bronchial biopsies were cut into small pieces and airway epithelial cells were isolated through subsequent cycles of enzymatic digestion with 0.05% trypsin—0.01% EDTA (Gibco) at 37 °C for a total time length of about 2 h. Isolated cells were cultured at a density of 8000 cells/cm^2^ in BEGM™ medium containing Bronchial Epithelial Basal Medium (BEBM) supplemented with bovine pituitary extract, insulin, hydrocortisone, gentamicin/amphotericin-B, retinoic acid, transferrin, triiodothyronine, epinephrine and human epidermal growth factor as described in the BEGM™, Lonza datasheet. Cultures were fed every other day and serially expanded for seven passages after reaching sub-confluent conditions, to evaluate their growth potential, population doubling and doubling time.

The population doubling was calculated as 3.32*(log_10_ N2—log_10_ N1), where N2 and N1 indicate respectively the number of cells at sub-confluence and the number of seeded cells. Instead, the doubling time was calculated as the ratio between the time to reach the sub-confluence (hours) and the corresponding population doubling.

Cells at different passages (three, six and nine) were cultured over glass coverslips until sub-confluence or for 30 days (cells at passage three) before undergoing immunofluorescence staining (see below).

### Colony-forming efficiency assay

For the colony-forming efficiency (CFE) assay, a small aliquot of cells (ranging from 500 to 2000), either directly isolated from human and swine airway biopsies or cultured for one passage as described above, was plated onto indicator culture dishes previously coated with lethally irradiated 3T3-J2 cells as feeder layer. Cells were cultured in Dulbecco’s modified Eagle’s (DMEM) and Ham’s F12 media (2:1 mixture) containing fetal bovine serum (FBS) (10%), insulin (5 µg/ml), adenine (0.18 mM), hydrocortisone (0.4 µg/ml), cholera toxin (0.1 nM), triiodothyronine (Liothyronine Sodium) (2 nM), glutamine (4 mM), penicillin–streptomycin (50 lU/ml), and epidermal growth factor (EGF, 10 ng/ml). Colonies were fixed 12 days later and stained with Rhodamine B (Sigma-Aldrich, St. Louis, Missouri, USA). Under examination with a dissecting microscope, colonies were scored as progressively growing or aborted, as previously described^24,25^. CFE values were calculated as the ratio of the number of grown colonies to the number of plated cells, whereas the percentage of aborted colonies was obtained from the ratio between abortive colonies and the number of all colonies grown in the indicator dish.

### Airlifted cultures

Tracheal and bronchial epithelial cells derived from human and porcine specimens were cultured in Air–Liquid Interface (ALI) conditions for about 30 days to allow complete differentiation of airway epithelial cells. Briefly, Millicell® Culture Plate Inserts (Merck Millipore) were coated with human fibronectin (Sigma Aldrich) at 17 μg/cm^2^, incubated overnight at room temperature and air dried. Airway human and porcine epithelial cells were then seeded onto Millipore inserts (133.000 cells/cm^2^) and cultivated in submerged condition with BEGM culture medium until cells reached the confluence. Then, BEGM culture medium was removed from the apical and basal chambers and Bronchial Air–Liquid Interface Medium (B-ALI™ from Lonza, specifically defined for human bronchial/tracheal epithelial cells) was added to the basal chambers only to start the air–liquid interface cultures. B-ALI Bronchial Air–Liquid Interface Medium was composed by B-ALI differentiation medium supplemented with bovine pituitary extract, insulin, hydrocortisone, gentamicin/amphotericin, retinoic acid, transferrin, triiodothyronine, epinephrine, human epidermal growth factor and B-ALI inducer as described in the related datasheet. The culture medium was changed every other day and regenerated epithelia were used for trans-epithelial electric resistance (TEER) measurement as well as for embedding in an optimal cutting temperature compound (OCT) to be stained by immunofluorescence.

### Trans-epithelial electrical resistance measurement

Transepithelial electrical resistance (TEER) was measured for human and porcine airway epithelia cultured on Millicell® inserts in ALI condition on days 3, 9, 15, 19, 22, and 27. TEER readings were obtained using a Millicell® ERS-2 voltmeter (Electrical Resistance System, Merck Millipore). The apical surface of cultured epithelia was submerged with B-ALI™ only for the time needed to perform the measurement. Normalization of replicates’ resistance values was performed by subtracting the corresponding value of TEER measured in control inserts that did not contain cells. The data were calculated as means ± SD of the measurements performed at each time point.

### Immunofluorescence staining

For the Immunofluorescence (IF) analysis, 10 μm-thick sections of tracheal and bronchial OCT-embedded samples were cut with a cryostat, mounted onto glass slides and fixed with 3% paraformaldehyde (PFA) for 10 min at room temperature (RT) or cold methanol for 10 min at -20 °C. Tracheal and bronchial cells cultured onto glass slides were washed with phosphate buffered saline (PBS 1X) solution and fixed with 3% PFA (10 min at RT) or cold methanol (10 min at -20 °C). Fixed cells were then permeabilized with 0.5%—1% Triton X100 (Biorad) in PBS 1X (10 min at RT), blocked with BSA 2%, incubated with the primary antibodies 30 min at 37 °C (all primary antibodies except for anti-Ki67, incubated overnight at + 4 °C), and thereafter with the corresponding secondary antibodies (30 min at 37 °C). The nuclei were stained with 4',6 diamidino-2-phenylindole (DAPI) and the slide were mounted with Fluorescent Mounting Medium (Dako, Agilent Technologies). Immunofluorescence images were acquired using LSM 900 confocal microscope (Zeiss) and Zen 3.3 software (Zeiss). Cytokeratin 5 (1:1000; Biolegend), Cytokeratin 7 (1:100; Progen), Cytokeratin 14 (1:5000; Biolegend), Ki67 (1:50; Invitrogen), p75NTR (1:50; Cell Signalling), ZO-1 (1:100; Invitrogen), MU5AC (1:50, Progen), and Acetylated $\mathrm{\alpha }$-Tubulin (1:100; Sigma) antibodies were used for immunofluorescence staining.

### Scanning electron microscopy (SEM)

The cultures were fixed in 2.5% (v/v) glutaraldehyde in 0.1 M sodium cacodylate for 1 h at 4 °C, washed with 0.1 M sodium cacodylate, postfixed in 1% (w/v) OsO_4_ for 1 h at room temperature. Samples were dehydrated in an ascending series of ethanol and critical point dried through liquid carbon dioxide. Samples were mounted on aluminium stubs and coated with a gold sputter. Samples were observed under a NOVA NanoSEM 450 and images were acquired in high-vacuum mode with TLD detector operated at 8 kV.

### Statistical analysis

The results are presented as mean ± standard deviation (SD). The significance of differences was analysed using Prism 9 software (version 9.4.1, GraphPad Software, San Diego, CA, USA) by Student’s unpaired two-sample t-test and p < 0.05 was considered significant.

### Supplementary Information

Supplementary Information.

## Data Availability

All data generated or analysed during this study are included in this published article and its supplementary information files.

## References

[1] Sceberras V (2020). Preclinical study for treatment of hypospadias by advanced therapy medicinal products. World J. Urol..

[2] Ribitsch I (2020). Large animal models in regenerative medicine and tissue engineering: To do or not to do. Front. Bioeng. Biotechnol..

[3] Perel P (2007). Comparison of treatment effects between animal experiments and clinical trials: Systematic review. Br. Med. J..

[4] Prabhakar S (2012). Translational research challenges: Finding the right animal models. J. Invest. Med..

[5] Sykes M, Sachs DH (2019). Transplanting organs from pigs to humans. Sci. Immunol..

[6] Mak IWY, Evaniew N, Ghert M (2014). Lost in translation: Animal models and clinical trials in cancer treatment. Am. J. Transl. Res..

[7] Sachs DH (1994). The pig as a potential xenograft donor. Vet. Immunol. Immunopathol..

[8] Meurens F, Summerfield A, Nauwynck H, Saif L, Gerdts V (2012). The pig: A model for human infectious diseases. Trends Microbiol..

[9] Aigner B (2010). Transgenic pigs as models for translational biomedical research. J. Mol. Med..

[10] Swindle MM, Makin A, Herron AJ, Clubb FJ, Frazier KS (2012). Swine as models in biomedical research and toxicology testing. Vet. Pathol..

[11] Segatto NV (2017). The oncopig cancer model as a complementary tool for phenotypic drug discovery. Front. Pharmacol..

[12] Perleberg C, Kind A, Schnieke A (2018). Genetically engineered pigs as models for human disease. DMM Dis. Models Mech..

[13] Lu T, Yang B, Wang R, Qin C (2020). Xenotransplantation: Current status in preclinical research. Front. Immunol..

[14] Rehmani SS (2017). Three-dimensional-printed bioengineered tracheal grafts: Preclinical results and potential for human use. Ann. Thorac. Surg..

[15] Niermeyer WL, Rodman C, Li MM, Chiang T (2020). Tissue engineering applications in otolaryngology—The state of translation. Laryngosc. Invest. Otolaryngol..

[16] Judge EP (2014). Anatomy and bronchoscopy of the porcine lung: A model for translational respiratory medicine. Am. J. Respir. Cell Mol. Biol..

[17] Wiseman LR, Bryson HM (1994). Porcine-derived lung surfactant. Drugs.

[18] Rogers CS (2008). The porcine lung as a potential model for cystic fibrosis. Am. J. Physiol. Lung Cell Mol. Physiol..

[19] Meyerholz DK, Reznikov LR (2022). Influence of SARS-CoV-2 on airway mucus production: A review and proposed model. Vet. Pathol..

[20] Jia Y (2019). Phenotypic analysis of BrdU label-retaining cells during the maturation of conducting airway epithelium in a porcine lung. Stem Cells Int..

[21] Liu X (2007). Bioelectric properties of chloride channels in human, pig, ferret, and mouse airway epithelia. Am. J. Respir. Cell Mol. Biol..

[22] Go T (2010). Both epithelial cells and mesenchymal stem cell-derived chondrocytes contribute to the survival of tissue-engineered airway transplants in pigs. J. Thorac. Cardiovasc. Surg..

[23] Rock, J. R. *et al. Basal Cells as Stem Cells of the Mouse Trachea and Human Airway Epithelium*. www.pnas.org/cgi/content/full/ (2009). 10.1073/pnas.0906850106 PMC2714281 19625615

[24] Barrandon Y, Green H (1987). Three clonal types of keratinocyte with different capacities for multiplication. Proc. Natl. Acad. Sci. USA.

[25] Pellegrini G (1999). Location and clonal analysis of stem cells and their differentiated progeny in the human ocular surface. J. Cell Biol..

[26] Corradini F (2016). Comparative assessment of cultures from oral and urethral stem cells for urethral regeneration. Curr. Stem Cell Res. Ther..

[27] Cole BB (2010). Tracheal basal cells: A facultative progenitor cell pool. Am. J. Pathol..

[28] Gerovac BJ (2014). Submersion and hypoxia inhibit ciliated cell differentiation in a Notch-dependent manner. Am. J. Respir. Cell Mol. Biol..

[29] Ware LB (2008). Modeling human lung disease in animals. Am. J. Physiol. Lung Cell Mol. Physiol..

[30] Stennert E, Siefer O, Zheng M, Walger M, Mickenhagen A (2008). In vitro culturing of porcine tracheal mucosa as an ideal model for investigating the influence of drugs on human respiratory mucosa. Eur. Arch. Oto-Rhino-Laryngol..

[31] Wang H (2018). Establishment and comparison of air-liquid interface culture systems for primary and immortalized swine tracheal epithelial cells. BMC Cell Biol..

[32] Khoufache K (2010). Primary in vitro culture of porcine tracheal epithelial cells in an air-liquid interface as a model to study airway epithelium and Aspergillus fumigatus interactions. Med. Mycol..

[33] Bateman AC (2010). Glycan analysis and influenza A virus infection of primary swine respiratory epithelial cells: The importance of NeuAcα2-6 glycans. J. Biol. Chem..

[34] Sreenivasan CC (2019). Development and characterization of swine primary respiratory epithelial cells and their susceptibility to infection by four influenza virus types. Virology.

[35] Wu NH (2016). The differentiated airway epithelium infected by influenza viruses maintains the barrier function despite a dramatic loss of ciliated cells. Sci. Rep..

