# Supplementary material for: Validation of airway porcine epithelial cells as an alternative to human in vitro preclinical studies
Source: Sci Rep. 2023 Sep 28;13:16290. doi: 10.1038/s41598-023-43284-7 (PMC10539525; doi:10.1038/s41598-023-43284-7)
Supplement: Supplementary file 1 — Supplementary Information. [file 41598_2023_43284_MOESM1_ESM.pdf]

Supplementary Information File

Validation of airway porcine epithelial cells as an alternative to human *in vitro* preclinical studies

Vincenzo Giuseppe Genna[1,2*+], Davide Adamo[1+], Giulia Galaverni[1], Fabio Lepore[1], Federica Boraldi[3], Daniela Quaglino[3], Filippo Lococo[4,5], Graziella Pellegrini[1,2*]

1 Centre for Regenerative Medicine "Stefano Ferrari", University of Modena and Reggio Emilia, Modena, Italy.
2 Holostem Terapie Avanzate s.r.l., Modena, Italy.
3 Department of Life Sciences, University of Modena and Reggio Emilia, Modena, Italy.
4 Thoracic Surgery Unit, Fondazione Policlinico Universitario A. Gemelli IRCCS, Rome, Italy.
5 Università Cattolica del Sacro Cuore, Rome, Italy.
[*] email: vg.genna@gmail.com; graziella.pellegrini@unimore.it
[+]these authors contributed equally to this work

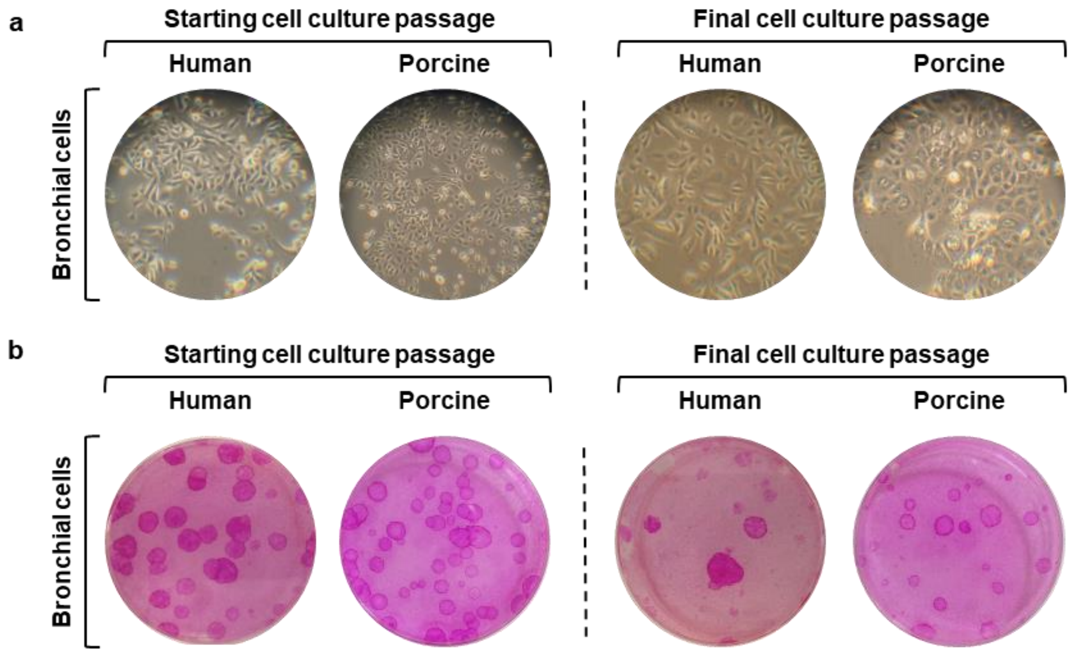

Supplementary Figure S1. Cell morphology and clonogenic potential evaluation of primary airway epithelial cells. (a) Representative images of human and porcine bronchial epithelial cell cultures at starting (pII) and final (pIX) analysed passages. (b) CFE assay performed to evaluate the number of clonogenic and aborted colonies at starting and final sub-cultures.

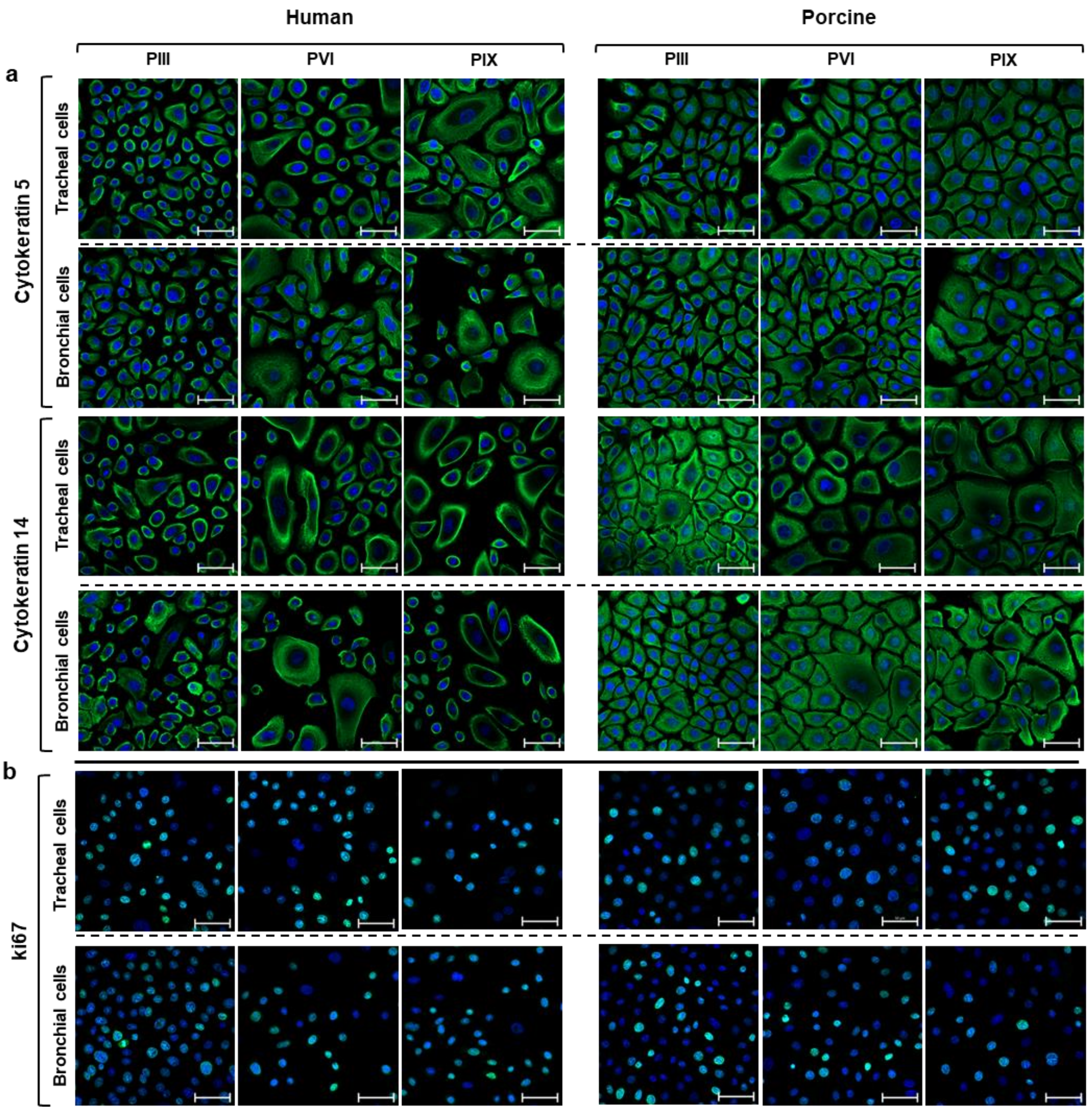

Supplementary Figure S2. Analysis of cytokeratins and proliferative cell marker expression in *in vitro* cultured airway epithelial cells. (a) Human and porcine tracheal and bronchial epithelial cells at the starting (pIII), middle (pVI), and late (pIX) cell culture passages were stained for epithelial-specific cell markers, namely cytokeratins. Basal CK5 and CK14 (green) expression was comparable in all the analysed samples. (b) Human and porcine airway epithelial cells showed a similar ki67 (green) expression throughout the serial passages, suggesting a comparable proliferative potential between the two species. Nuclei were counterstained with DAPI, scale bar = 50µm. p = passage
